# Effects of Inhibiting CoQ_10_ Biosynthesis with 4-nitrobenzoate in Human Fibroblasts

**DOI:** 10.1371/journal.pone.0030606

**Published:** 2012-02-16

**Authors:** Catarina M. Quinzii, Saba Tadesse, Ali Naini, Michio Hirano

**Affiliations:** 1 Department of Neurology, Columbia University Medical Center, New York, New York, United States of America; 2 Department of Pathology and Cell Biology, Columbia University Medical Center, New York, New York, United States of America; Hospital Vall d'Hebron, Spain

## Abstract

Coenzyme Q_10_ (CoQ_10_) is a potent lipophilic antioxidant in cell membranes and a carrier of electrons in the mitochondrial respiratory chain. We previously characterized the effects of varying severities of CoQ_10_ deficiency on ROS production and mitochondrial bioenergetics in cells harboring genetic defects of CoQ_10_ biosynthesis. We observed a unimodal distribution of ROS production with CoQ_10_ deficiency: cells with <20% of CoQ_10_ and 50–70% of CoQ_10_ did not generate excess ROS while cells with 30–45% of CoQ_10_ showed increased ROS production and lipid peroxidation. Because our previous studies were limited to a small number of mutant cell lines with heterogeneous molecular defects, here, we treated 5 control and 2 mildly CoQ_10_ deficient fibroblasts with varying doses of 4-nitrobenzoate (4-NB), an analog of 4-hydroxybenzoate (4-HB) and inhibitor of 4-para-hydroxybenzoate:polyprenyl transferase (COQ2) to induce a range of CoQ_10_ deficiencies. Our results support the concept that the degree of CoQ_10_ deficiency in cells dictates the extent of ATP synthesis defects and ROS production and that 40–50% residual CoQ_10_ produces maximal oxidative stress and cell death.

## Introduction

Ubiquinone or coenzyme Q (CoQ) is a lipophilic molecule present in virtually all cell membranes. Essential for multiple metabolic processes, CoQ is required for antioxidant defenses and electron transport from complex I and II to complex III in the mitochondrial respiratory chain [1]–[3]. CoQ is synthesized within mitochondria and is composed of a benzoquinone ring and a polyprenyl side chain. The length of the isoprenoid in ubiquinone varies among species; the predominant form in human is composed of 10 isoprenyl units and is designated CoQ_10_. Current knowledge about CoQ biosynthetic pathway in eukaryotes is mainly derived from studies of *S. cerevisiae* [4], [5]. At least 10 complementation groups of Q mutant yeast have been identified [6]–[8]. Whereas the quinone ring is derived from tyrosine or phenylalanine, the isoprenoid side chain is generated by addition of isopentenyl diphosphate molecules, derived from the mevalonate pathway, to farnesyl diphosphate in multiple steps catalyzed by polyprenyl diphosphate synthase (in human, a heterotetramer of two protein subunits, PDSS1 and PDSS2) [9]. Decaprenyl diphosphate and para-hydroxybenzoate (PHB) are condensed in a reaction catalyzed by 4-hydroxybenzoate:polyprenyl transferase or COQ2, and the benzoate ring is then modified by at least six enzymes, which catalyze methylation, decarboxylation, and hydroxylation reactions to synthesize CoQ_10_ [4], [5], [10]. ADCK3 is a protein kinase involved in CoQ biosynthesis and its yeast homolog, Coq8p, is required for the formation or maintenance of the multisubunit Q-biosynthetic complex and phosphorylation of Coq3p, Coq5p, and Coq7p [11]–[16].

Although CoQ_10_ deficiency has been identified in more than 100 patients with a wide spectrum of phenotypes, the molecular genetic bases have been identified in a minority of the patients and the pathophysiological consequences of human CoQ_10_ deficiency at the cellular level remain largely unknown [17]–[19].

In previous studies, we investigated the consequences of varying degrees of CoQ_10_ deficiency on ROS production, mitochondrial functions, and cell viability in skin fibroblasts with CoQ_10_ deficiency due to different molecular defects including mutations in *COQ2* [20], *PDSS2* [21]–[23], *ADCK3* (*CABC1*) [12], and *COQ9* [24]. We reported that cultured fibroblasts with severe CoQ_10_ deficiency (<20% of normal) have marked bioenergetic defects without significant oxidative stress, whereas intermediate CoQ_10_ deficiency (30–45% of normal) causes moderate bioenergetic defects but marked increases in ROS production, lipid oxidation, and cell death [2], [3]. Not surprisingly, cells with mild CoQ_10_ deficiency (>60% of normal) did not show increased ROS production or oxidative damage. However, because we compared cultured fibroblasts with diverse molecular genetic defects, factors other than CoQ_10_ deficiency may have contributed to their differing *in vitro* phenotype. Therefore, to assess the role of CoQ_10_ level on mitochondrial bioenergetics impairment, oxidative stress, and cell death in a uniform genetic background, we treated multiple cell lines with increasing dosages of 4-nitrobenzoate, which inhibits 4-hydroxybenzoate:polyprenyltransferase (COQ2) leading to dose-dependent decreases of CoQ in mammalian cells without directly inducing oxidative stress or mitochondrial respiration impairment [25].

## Methods

### Cell culture

Mitochondrial bioenergetic and oxidative stress experiments were performed in 5 control skin fibroblasts cell lines with normal CoQ_10_ levels and in 2 skin fibroblasts cell lines with *ADCK3* mutations, P1 (p.Y514C and p.T584del) and P2 (homozygous p.Q167LfxX36), previously demonstrated to have defects of ubiquinone biosynthesis, measured by incorporation of radiolabeled parahydroxybenzoate (^14^C-PHB) (450 Ci/mol) [2], [20].

Cells were grown in Dulbecco's minimum essential medium (DMEM) supplemented with 10% fetal bovine serum (FBS), 5 ml MEM vitamins, 5 ml MEM non-essential amino acids, 1 ml fungizone, and 5 ml penicillin-streptomycin until 50% confluent.

Experiments were performed after 6 days of incubation in RPMI 1640 glucose-free medium with 10% regular FBS, 25 mM HEPES, 1.5 mM Glutamax, 25 mM galactose, 1 ml fungizone, and 5 ml penicillin-streptomycin supplemented with one of the following: 4 mM DMSO, 1 mM 4-NB, 2 mM 4-NB, 3 mM 4-NB, or 4 mM 4-NB. To demonstrate that the effects of 4 mM 4-NB were caused by CoQ_10_ deficiency rather than side effects of the compound, control cell lines were supplemented also with 4-NB+2 mM 4-HB and 4-NB+ 5 µM CoQ_10_ [25], [26].

We performed experiments in galactose-medium because we previously demonstrated that CoQ_10_ deficient fibroblasts do not manifest increased oxidative stress and cell death or reduced mitochondrial function when cultured in glucose-rich medium [3]. *ADCK3* mutant skin fibroblasts manifest mitochondrial bioenergetics impairment and oxidative stress when cultured in galactose RPMI 1640 media with dialyzed FBS, but not in galactose RPMI media with undialyzed FBS because glucose in FBS allows anerobic glycolysis to maintain the cellular energy charge [2]. In contrast, the slow metabolism of galactose to glucose-1-phosphate is insufficient for glycolytic synthesis of ATP oxidative phosphorylation is impaired [27].

Medium was changed at day 1, 3 and 5, and cells were collected at day 7 [25]. 4-NB and 4-HB (Sigma-Aldrich, St. Louis MO, USA) were dissolved in DMSO and stored at −20°C in 0.1 M stock solutions. All other cell culture reagents were obtained from Invitrogen (Invitrogen Corp., Eugene, OR, USA).

All cell lines at passage 7–10 were cultured at least 3 times, therefore, each value in the results represents the mean of at least 3 measurements.

### CoQ_10_ levels

CoQ_10_ in fibroblasts was extracted in hexane:ethanol mixture. The lipid component of the extract was separated by high-performance liquid chromatography (HPLC) on a reverse phase Symmetry® C18 3.5 mm, 4.6×150 mm column (Waters), using a mobile phase consisting of methanol, ethanol, 2-propanol, acetic acid (500∶500∶15∶15) and 50 mM sodium acetate at a flow rate of 0.9 ml/min. The electrochemical detection system consisted of an ESA Coulochem III with a guard cell (upstream of the injector) at +900 mV, conditioning cell at 600 mV (downstream of the column), followed by the analytical cell at +500 mV. CoQ_10_ concentration was estimated by comparison of the peak area with those of standard solutions of known concentrations [26].

### Adenine nucleotides levels

To determine levels of adenine nucleotides, cells were washed in ice-cold phosphate-buffered saline (PBS) and then collected in ice-cold 0.5 M perchloric acid using a scraper. After centrifugation at 3,000 *g* for 3 min at 4°C, pellets were suspended in 200 µl of ice-cold 0.5 M perchloric acid, vortexed for 30 s, and centrifuged at 11,000 *g* for 10 min at 4°C. The pellets were stored at −80°C for protein measurement. Adenine nucleotides in supernatants were measured in an Alliance HPLC (Waters Corporation, Milford, MA, USA) with an Alltima C18-NUC HPLC reverse-phase column (Alltech Associates, Deerfield, IL, USA) [3]. Adenine nucleotide levels were expressed in nmol/mg protein [26].

### Mitochondrial membrane potential

To estimate mitochondrial membrane potential, control and mutant cells were exposed to tetramethylrhodamine ethyl ester (TMRE) (Molecular Probes, Invitrogen Corp., Eugene, OR, USA). Approximately 1×10^6^ cells were trypsinized, incubated with TMRE for 20 min at 37°C, washed twice with PBS, re-suspended in 500 µl of PBS and kept in ice.

Cytofluorometric analysis of stained cells was performed on a FACSCalibur. Data were acquired using Cell Pro Quest and analyzed using Flowjo software (Becton Dickinson, NJ, USA) [2].

### Oxidative stress analyses

To estimate production of ROS, control and mutant cells were exposed to MitoSOX Red, a fluorochrome specific for anion superoxide produced in the inner mitochondrial compartment (Molecular Probes, Invitrogen Corp., Eugene, OR, USA). Approximately 1×10^6^ cells were trypsinized, incubated with MitoSox for 20 min at 37°C, washed twice with PBS and resuspended in 500 µl of PBS. Cytofluorometric analysis was performed on a FACSCalibur. Data were acquired using Cell Pro Quest and analyzed using Flowjo software (Becton Dickinson, NJ, USA) [3].

To assess oxidative damage, we assessed lipid peroxidation (LPO) after 4 mM 4-NB supplementation. For LPO measurements, confluent cells were collected in PBS from 15 cm plates using scrapers. After centrifugation at 3,000 *g* for 5 min, cells were suspended in 20 mM Tris–HCl buffer, pH 7.4, containing 5 mM butylated hydroxytoluene, and sonicated to lyse the cell. To remove large particles, the samples were centrifuged at 3,000 *g* for 10 min at 4°C. Aliquots of the supernatants were either stored at −80°C for total protein determination or used for LPO. Bioxytech LPO-568 assay kit was used to determine both malondialdehyde (MDA) and 4-hydroxyalkenals (4HE) (Oxis International, Foster City, CA, USA). Concentrations of LPO were normalized per mg protein [3].

### Cell death studies

Cell viability was monitored by trypan blue exclusion. Numbers of living and dead cells were determined using the Countess Automated Cell Counter (Invitrogen). Healthy nuclei from viable cells appeared round and phase bright, whereas nuclei from dead or dying cells appeared blue and irregularly shaped. All cells were counted and results were expressed as the ratio of living versus total cells.

### Statistical analysis

Control data are expressed as the mean ± standard deviation (SD) of 5 different normal fibroblast lines each analyzed in triplicate. Patients' data are expressed as the mean ± SD of triplicates experiments. Unpaired Student's T test with Welch correction was used. A p-value <0.05 was considered to be significant.

## Results

### CoQ_10_ level

**Figure 1:**
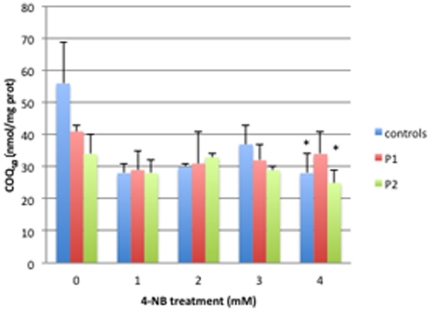
CoQ_10_ levels in control (n = 5), P1 and P2 skin fibroblasts after 4-NB treatment. CoQ_10_ levels are nmol/mg-protein (*P<0.05 vs. controls).

Baseline levels of CoQ_10_ were reduced in *ADCK3* mutant P1 (63%) and P2 (51%) relative to control fibroblasts (n = 5). Control and P2 fibroblasts showed decreased CoQ_10_ levels after 4 mM 4-NB supplementation and trends towards decreased levels after 1 mM, 2 mM, and 3 mM 4-NB treatment. In contrast, P1 fibroblasts showed a trend towards reduced CoQ_10_ levels after 1–4 mM 4-NB supplementation (Fig. 1).

**Figure 2:**
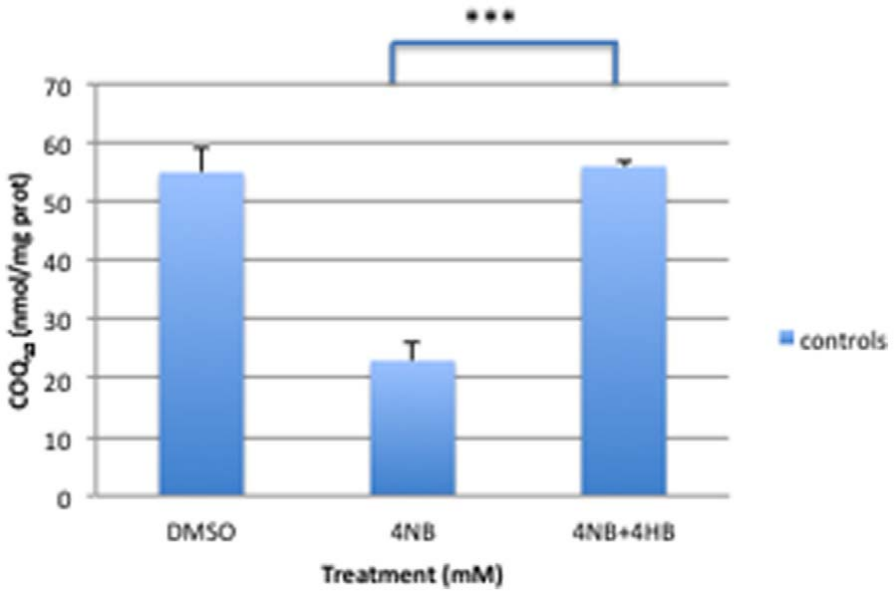
CoQ_10_ levels in control skin fibroblasts (n = 5) after treatment with DMSO, 4 mM 4-NB alone, or 4 mM 4-NB+2 mM 4-HB. The values are nmol/mg-protein (***P<0.001).

**Figure 3:**
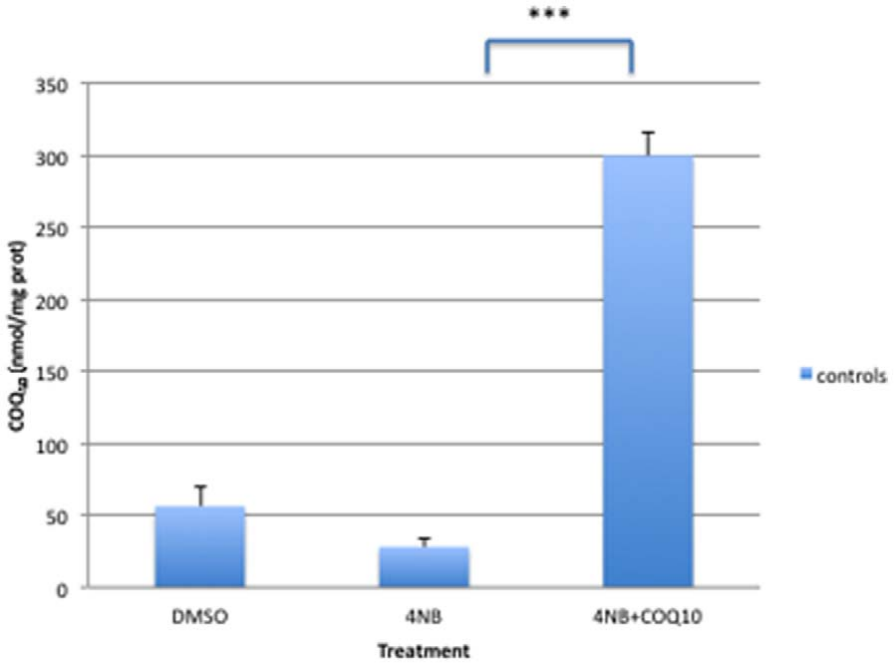
CoQ_10_ levels in control skin fibroblasts (n = 5) after treatment with DMSO, 4 mM 4-NB alone, or 4 mM 4-NB+5 µM CoQ_10_. The values are nmol/mg-protein (***P<0.001).

Co-treatment of control fibroblasts with 4 mM 4-NB and 2 mM 4-HB completely restored CoQ_10_ levels (Fig. 2) . Co-treatment of control fibroblasts with 4 mM 4-NB and 5 µM CoQ_10_ significantly increased levels of CoQ_10_ (Fig. 3).

### Adenine nucleotide levels

**Figure 4:**
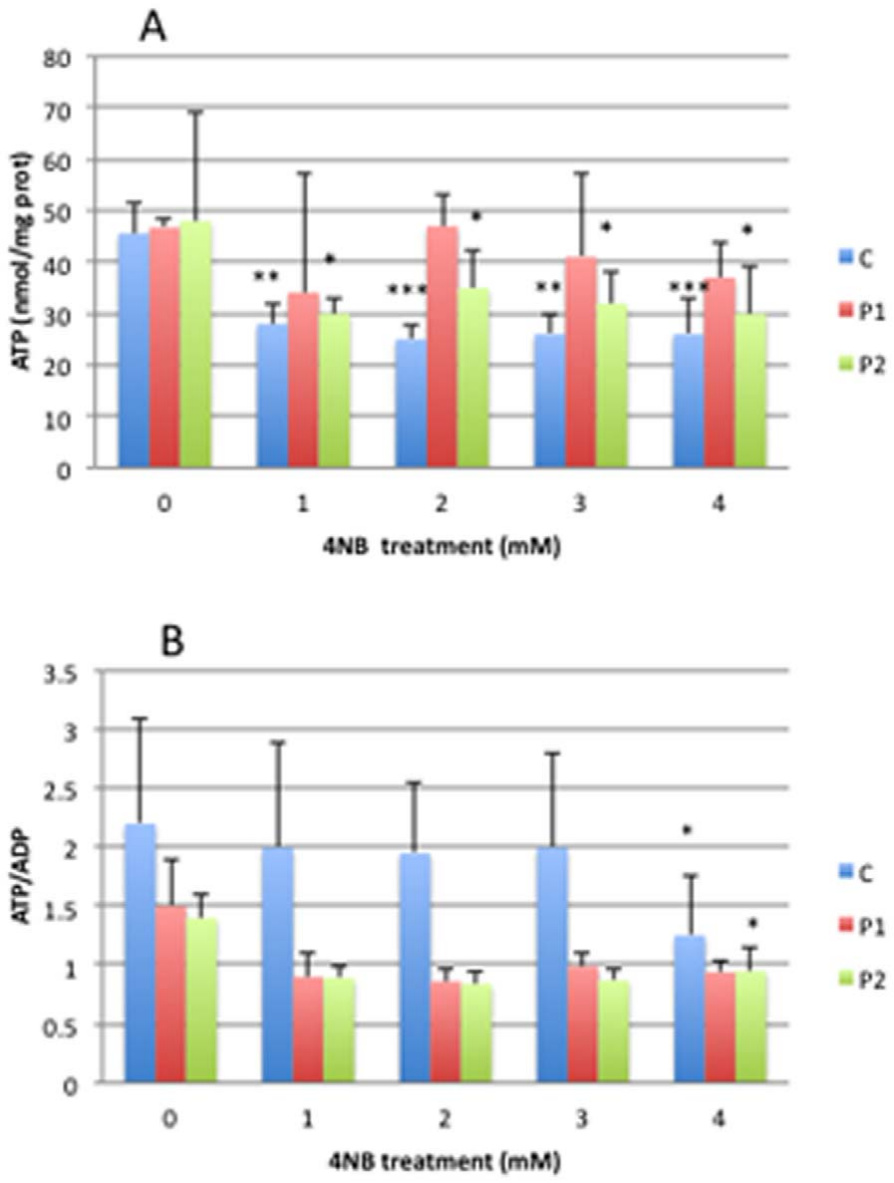
Adenine nucleotides levels in control (n = 5), P1 and P2 skin fibroblasts after 4-NB treatment (ATP in panel A and ATP/ADP in panel B). The values are nmol/mg-protein, P1 and P2 after 4 mM DMSO treatment. (* *P*<0.05 vs. controls; **P<0.01 vs. controls; ***P<0.001 vs. controls).

**Figure 5:**
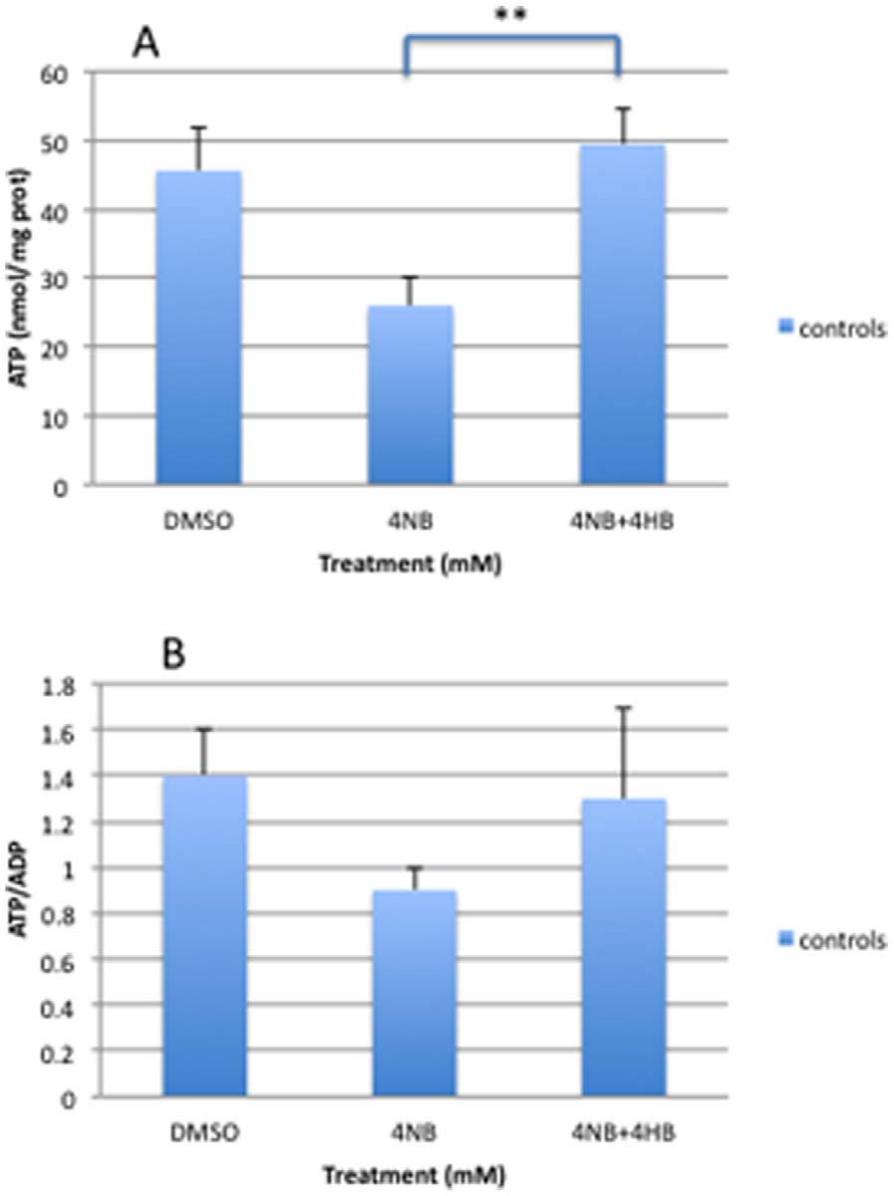
Adenine nucleotides levels in control skin fibroblasts (n = 5) after co-treatment with 4 mM 4-NB+2 mM 4-HB (ATP in panel A and ATP/ADP in panel B). The values are nmol/mg-protein (** *P*<0.01 vs. controls; ***P<0.001).

ATP level was significantly decreased in controls and P2 fibroblasts after 1 mM, 2 mM, 3 mM, and 4 mM 4-NB supplementation (Fig. 4A). P1 fibroblasts showed a trend towards reduction in ATP levels after 1–4 mM 4-NB supplementation (Fig. 4A). Control and P2 fibroblasts showed significant decreases in ATP/ADP ratio after 4 mM of 4-NB supplementation and trends towards decreased levels after 1 mM, 2 mM, and 3 mM 4-NB treatment (Fig. 4B). Co-treatment of control fibroblasts with 4 mM 4-NB and 2 mM 4-HB completely restored ATP level and ATP/ADP ratio (Fig. 5A and B).

### Mitochondrial membrane potential

**Figure 6:**
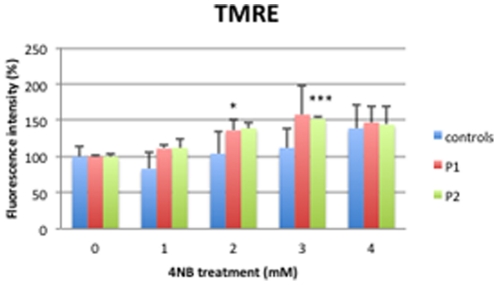
Assessment of mitochondrial membrane potential with TMRE in control (n = 5), P1 and P2 skin fibroblasts after 4-NB treatment. The values are expressed as percentage of controls, P1 and P2 after 4 mM DMSO treatment. (* *P*<0.05 vs. controls;***P<0.001 vs. controls).

**Figure 7:**
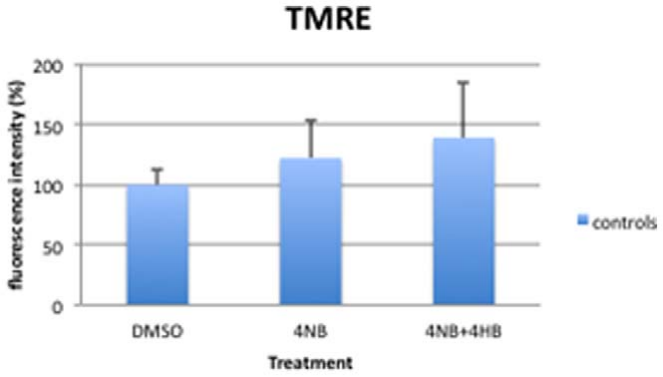
Assessment of mitochondrial membrane potential by TMRE in control skin fibroblasts (n = 5) after co-treatment with 4 mM 4-NB+2 mM 4-HB. The values are expressed as percentage of the control skin fibroblasts after 4 mM DMSO.

With 1 mM, 2 mM, and 3 mM 4-NB treatment, TMRE staining of control fibroblast was not significantly altered indicating stable mitochondrial membrane potential (Δ*Ψ*_m_), but a trend towards increased TMRE staining was observed with 4 mM 4-NB (Fig. 6). In contrast, after 2 mM, 3 mM, and 4 mM 4-NB supplementation, P1 and P2 cells revealed increased TMRE staining (Fig. 6). Co-treatment of control fibroblasts with 4 mM 4-NB and 2 mM 4-HB did not significantly alter TMRE intensity (Fig. 7).

### Oxidative stress analyses

**Figure 8:**
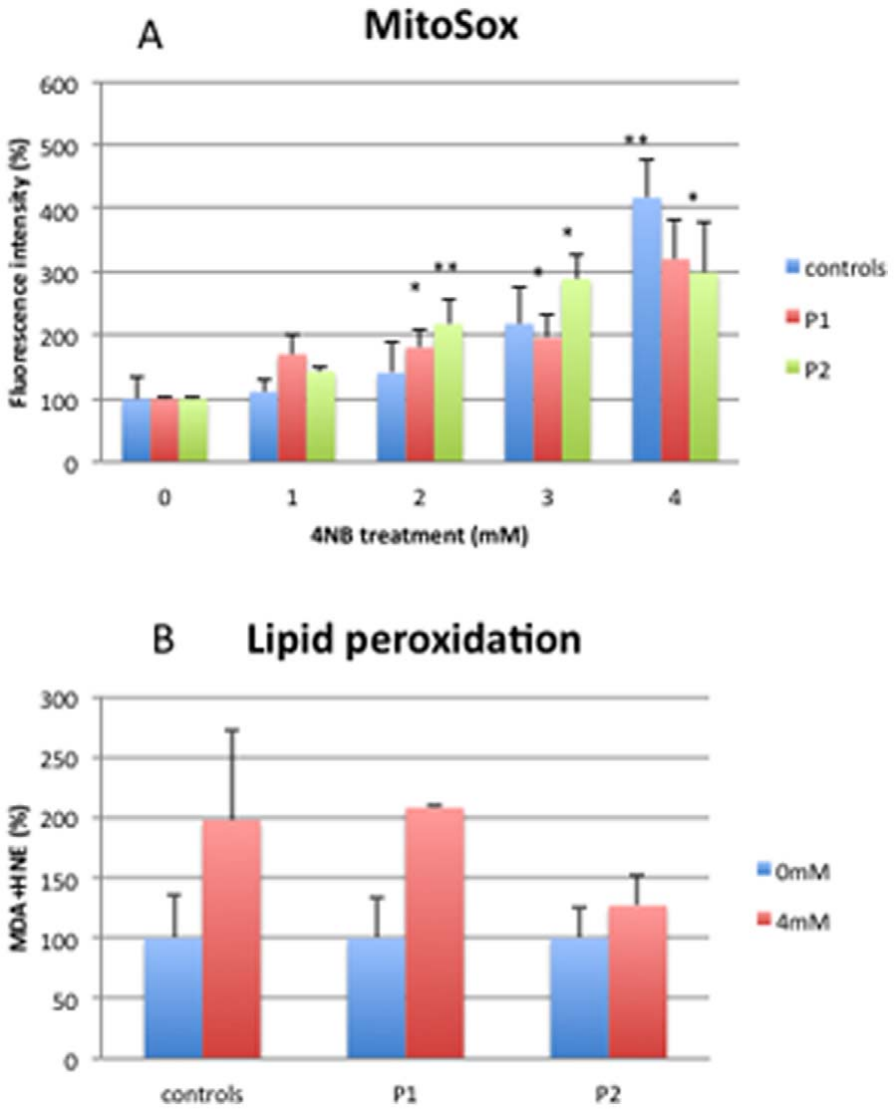
Quantitation of MitoSox staining by flow cytometry (panel A) and oxidation of lipids (panel B) in control (n = 5), P1 and P2 skin fibroblasts after 4-NB treatment. The values are expressed as percentage of controls, P1 and P2 after 4 mM DMSO treatment. (***P<0.001 vs. controls).

**Figure 9:**
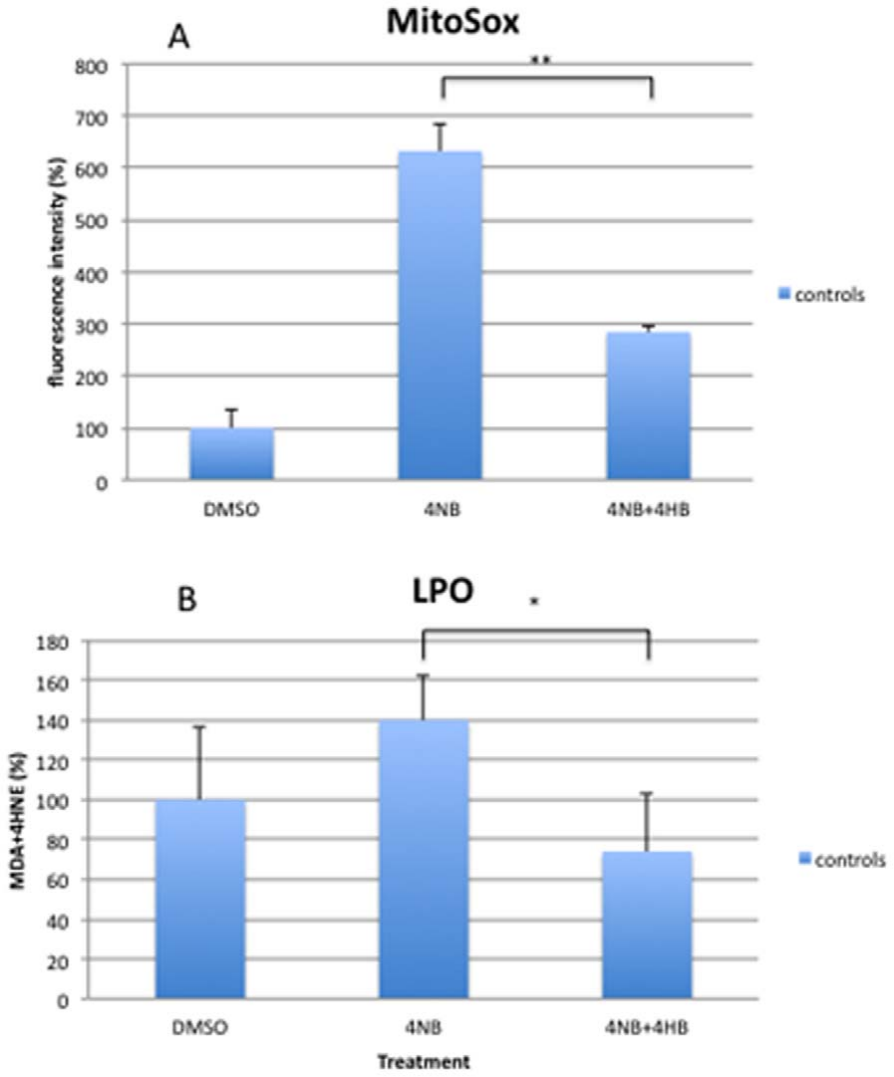
Quantitation of MitoSox staining by flow cytometry (panel A) and oxidation of lipids (panel B) in control skin fibroblasts (n = 5) after co-treatment with 4 mM 4-NB+2 mM 4-HB. The values are expressed as percentage of the control skin fibroblasts after 4 mM DMSO (** *P*<0.01).

Control fibroblasts showed significantly increased MitoSox staining after 3 mM and 4 mM 4-NB treatment while P1 and P2 fibroblasts showed significantly increased MitoSox staining after 2 mM, 3 mM, and 4 mM 4-NB treatment (Fig. 8A). Control fibroblasts treated with 4 mM 4-NB and 2 mM 4-HB showed significantly less MitoSox staining than cells treated with 4 mM 4-NB only (Fig. 9A).

Lipid peroxidation (LPO) was slightly increased in controls, P1 and P2 fibroblasts after 4 mM of 4-NB treatment (Fig. 8B). Control fibroblasts treated with 4 mM 4-NB and 2 mM 4-HB showed significantly less LPO than cells treated with only 4 mM 4-NB (Fig. 9B).

**Figure 10:**
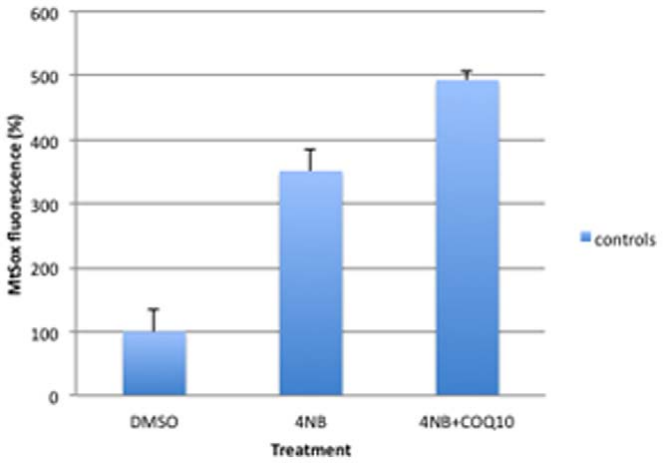
Quantitation of MitoSox staining by flow cytometry in control skin fibroblasts (n = 5) after co-treatment with 4 mM 4-NB+5 µM CoQ_10_. The values are expressed as percentage of the control skin fibroblasts after 4 mM DMSO (** *P*<0.01).

MitoSox staining of control fibroblasts treated with 4 mM 4-NB and 5 µM CoQ_10_ was not significantly different than staining in cells treated with 4 mM 4-NB only (Fig. 10).

### Cell death studies

**Figure 11:**
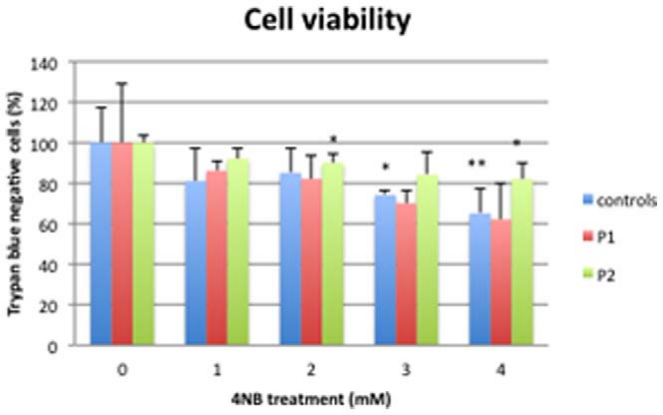
Trypan blue staining in control (n = 5), P1 and P2 skin fibroblasts after 4-NB treatment. The values are expressed as percentage of controls, P1 and P2 after 4 mM DMSO treatment. (* *P*<0.05 vs. controls; ** *P*<0.01 vs. controls).

Control fibroblasts showed significantly decreased cell viability after 3 mM 4-NB and 4 mM 4-NB while P1 and P2 cells showed decreased cell viability after 1 mM, 2 mm, 3 mM and 4 mM 4-NB treatments (Fig. 11).

**Figure 12:**
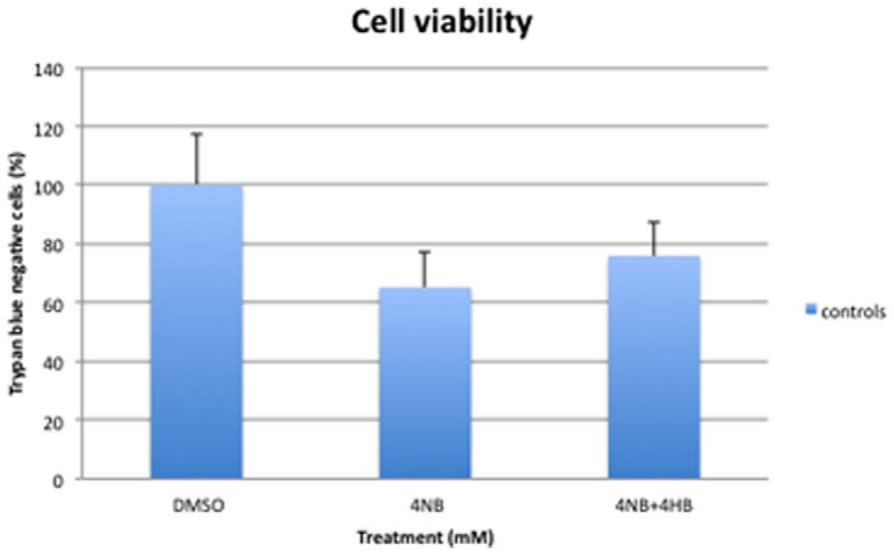
Quantitation of cell viability by trypan blue staining in control skin fibroblasts (n = 5) after co-treatment with 4 mM 4-NB+2 mM 4-HB. The values are expressed as percentage of the control skin fibroblasts after 4 mM DMSO.

**Figure 13:**
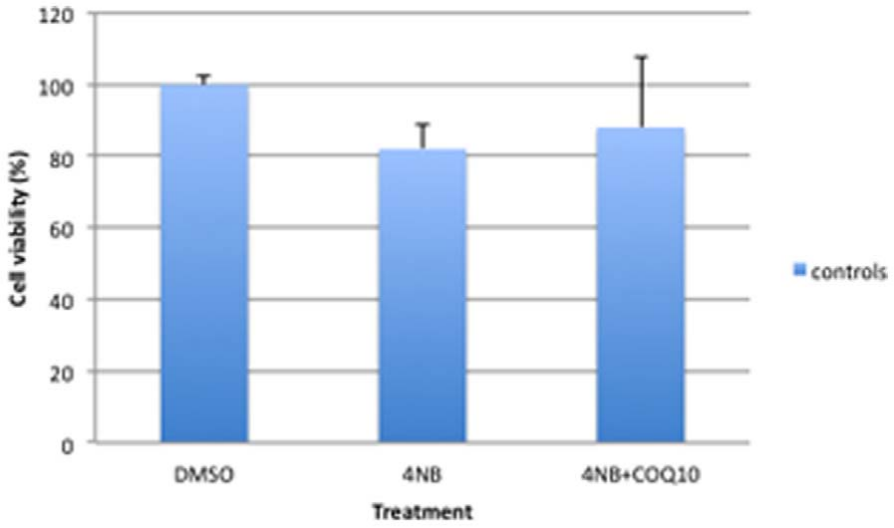
Trypan blue staining in control skin fibroblasts (n = 5) after co-treatment with 4 mM 4-NB+5 µM CoQ_10_. The values are expressed as percentage of the control skin fibroblasts after 4 mM DMSO. (* *P*<0.05 vs. controls; ** *P*<0.01 vs. controls).

Cell viability was normalized in control fibroblasts after co-treatment with 4 mM of 4-NB and 2 mM 4-HB (Fig. 12), but not after co-treatment with 4 mM 4-NB and 5 µM CoQ_10_ (Fig. 13).

## Discussion

Although a growing number of patients with CoQ_10_ deficiency due to a variety of mutations in CoQ_10_ biosynthetic genes has been reported [18], the pathophysiology of this syndrome is not well understood. Previous studies in human fibroblasts with decreased levels of CoQ_10_ indicated that different mechanisms might be involved. Studying cultured fibroblasts from two siblings with infantile-onset CoQ_10_-deficiency of unknown genetic etiology, Geromel and colleagues found mild respiratory chain defects without evidence of increased superoxide anions, lipid peroxidation, or apoptosis-mediated cell death [28]. We observed similar abnormalities in fibroblasts carrying mutations in *COQ9* and *PDSS2* [2], [3].

In contrast, Lopez-Martin and colleagues showed that fibroblasts from a patient with a homozygous *COQ2* mutation require uridine to maintain cell growth and proposed that deficiency of CoQ_10_ impaired pyrimidine biosynthesis due to dependence of dihydro-orotate dehydrogenase on ubiquinol [29]. In the same mutant *COQ2* cell lines, as well as in other two cell lines with CoQ_10_ deficiency and unknown molecular defects, evidence of autophagy and oxidative stress has been observed [22]. More recently the same group showed evidence of increase ROS production and autophagy in a cellular model of secondary CoQ_10_ deficiency due to the m.3243A>G mutation [30]. Intriguingly, ultrastructural evidence of autophagy has also been found in kidney of mice with a homozygous mutation in *Pdss2* [31]. Rapid improvements with supplemental CoQ_10_ or the antixoxidant probucol suggest that autophagy might be triggered by oxidative stress [32], [33].

Our previous studies of cells lines harboring *COQ2* mutations have indicated that CoQ_10_ level correlates with the production of ROS, and that oxidative stress plays an important role in the demise of *COQ2* mutant fibroblasts by activating cell-death related pathways, which are averted by antioxidant supplementation [3], [26]. Based on these observations, we hypothesized that the degree of CoQ_10_ deficiency in fibroblasts correlates with increased ROS production and cell death, independently of the primary molecular defect. In several mammalian cell lines other than human skin fibroblasts, 4-NB has been shown to decrease CoQ levels without any apparent direct toxic effects, in particular without inducing oxidative stress [25].

Here, we showed in control skin fibroblasts that pharmacological inhibition of COQ2 by 4-NB, leading to 40–50% residual CoQ_10_, is associated with increased oxidative stress and reduced viability, together with moderately decreased ATP levels and ATP/ADP ratio, similar to our observations in 3 different cell lines carrying mutations in COQ2.

Moreover, P1 and P2 *ADCK3* mutant cells, which have 51–63% residual CoQ_10_, do not show signs of oxidative stress at baseline, but showed significant increases in ROS (indicated by MitoSox staining) when they reached 40–50% residual CoQ_10_ levels, which occurs, not surprisingly, after lower doses (2–4 mM) of 4-NB than in control cell lines (4 mM 4NB). Intriguingly, wild-type control and *ADCK3* mutant cells reacted differently to 4-NB treatment. In control skin fibroblasts, we observed that only after maximum (4 mM) 4-NB treatment, CoQ_10_ content decreased significantly with proportional reductions in ATP level, whereas ROS production and trypan blue staining (indicating cell death) increased significantly after only 3 mM 4-NB. In cells with *ADCK3* mutations and decreased ubiquinone at baseline, 4-NB rapidly induced further reductions in CoQ_10_ levels, which plateaued at 40% of normal. Decreased CoQ_10_ concentration was associated with increased MitoSox and TMRE (indicating increased mitochondrial membrane potential) staining, and with slightly reduced cell viability while ATP levels were only mildly decreased. ATP/ADP ratio decreased starting from 1 mM 4-NB in both controls and mutant cells. Unexpectedly, we were unable to reduce CoQ_10_ levels below 40% of control mean, even in P1 and P2 cells, which had baseline CoQ_10_ deficiency. A pilot experiment of a control cell line treated with 8 mM 4-NB produced massive and irreversible cell death (data not shown), thus, higher doses of the compound could not be used to further decrease CoQ biosynthesis. The effects of the treatment were less prominent but were observed more rapidly in mutant cell lines relative to controls In support of variable response of cell lines to 4-NB are published observations that a human hepatocyte cell line (C3A cells) were less responsive to 4-NB-mediated decreases in CoQ as compared to rodent cell lines [25].

On one hand, CoQ_10_ deficiency appears to be deleterious in 4-NB treated fibroblasts because rescue by co-treatment of cells with 4-HB and 4-NB normalized CoQ_10_ levels, bioenergic defects, and oxidative stress. On the other hand, the compound may have toxic effects in addition to inhibition of CoQ biosynthesis because CoQ_10_ co-treatment did not rescue 4-NB effects and because of a floor-effect of 4-NB, which did not decrease CoQ_10_ level below 40% of normal. Failure of CoQ_10_ supplementation to rescue 4-NB toxicity is unlikely be due to inadequate penetration ubiquinone into mitochondria because we previously observed that incubation of ubiquinone-deficient fibroblasts with 5 µM CoQ_10_ for 1 week increases ATP levels and ATP/ADP ratios significantly, indicating normalization of the bioenergetic status and mitochondrial functions [2], [3].

We also reported that in both control and mutant cells, mitochondrial membrane potential (Δ*Ψ*_m_) was not reduced by low CoQ_10_ content, but rather Δ*Ψ*_m_ appeared to increase proportionally with increased ROS production and with decreased ATP levels and ATP/ADP ratios, supporting the hypothesis that early mitochondrial hyperpolarization might trigger mitochondrial ROS formation [2]. It is possible that the mitochondrial membrane potential is enhanced by the F(1)F(0) ATPase operating in ‘reverse’ mode, as suggested by other in vitro models of mitochondrial respiratory chain defects [34], [35].

Thus, our work in human skin fibroblasts with pharmacologically induced defects of ubiquinone biosynthesis have confirmed that increased ROS production contributes to the pathomechanism of CoQ_10_ deficiency associated with inhibition of *COQ2* and that partial CoQ_10_ deficiency (40–50% residual) is associated with increased ROS production, hyperpolarization, and cell death compared to CoQ_10_ defiency that is mild (>50% of normal) or severe (<30% of normal) [2], [3].

Our observations of deleterious oxidative stress in ubiquinone-deficient human fibroblasts are supported by studies of other *in vitro* and *in vivo* models of CoQ deficiency. ROS production was enhanced in the coq10 and coq2 mutant *S. cerevisiae* [36], [37] while coq7 and coq2 mutant *S. pombe* displayed hypersensitivity to hydrogen peroxide and a requirement for antioxidants for growth on minimal medium indicating a key pathogenic role of oxidative stress in yeast models of CoQ deficiency [38], [39]. RNAi of coq-1,coq-2, and coq-3 in *C. elegans* GABA neurons led to activation of cell death pathway featuring elements of apoptosis and necrosis [40], while, in *C. elegans* low CoQ levels causing respiratory chain defects was associated with low ROS production and life span extension [41]. Apoptosis was also observed in embryos of embryonically lethal coq7 defective mice [42]; and intracellular superoxide was significantly elevated in HL-60 cells treated with *p*-aminobenzoate, an inhibitor of COQ2 [43]. Furthermore, as noted above, the rapid improvement of *Pdss2* mutant mice with CoQ_10_ or probucol supplementation support the hypothesized role of increased ROS production in the pathogenesis of CoQ deficiency.

Our findings provide insights into the pathomechanisms underlying primary CoQ deficiency by demonstrating that the degree of bioenergetic defect and ROS production are related to the level of ubiquinone. Thus, depending on the severity of CoQ_10_ deficiency, biochemical targets for therapy may vary and may be relevant to other mitochondrial respiratory chain disorders. Moreover, the results may be germane to the pathogenesis and therapies of other neurodegenerative diseases with mitochondrial dysfunction.
